# Bottlenecks to clinical translation of direct brain-computer interfaces

**DOI:** 10.3389/fnsys.2014.00226

**Published:** 2014-12-02

**Authors:** Mijail D. Serruya

**Affiliations:** Department of Neurology, Thomas Jefferson UniversityPhiladelphia, PA, USA

**Keywords:** brain-computer interface, neurotechnology, device approval, commercialization, neuroprosthetic

## Abstract

Despite several decades of research into novel brain-implantable devices to treat a range of diseases, only two—cochlear implants for sensorineural hearing loss and deep brain stimulation for movement disorders—have yielded any appreciable clinical benefit. Obstacles to translation include technical factors (e.g., signal loss due to gliosis or micromotion), lack of awareness of current clinical options for patients that the new therapy must outperform, traversing between federal and corporate funding needed to support clinical trials, and insufficient management expertise. This commentary reviews these obstacles preventing the translation of promising new neurotechnologies into clinical application and suggests some principles that interdisciplinary teams in academia and industry could adopt to enhance their chances of success.

## Introduction

“Brain-computer interfaces” (BCI) and “brain-machine interfaces” (BMI) comprise a class of medical devices designed to restore independent function lost by neurological disease or injury. The qualifier “direct” implies that some component of the artificial device is physically implanted into the brain. While the BCI terminology usually refers to techniques that sense electrical activity in the brain to determine intended movement with the goal of restoring independent communication and movement in patients with paralysis, in principle any device implanted into the brain that includes electronic components could be considered a BCI, for example systems to detect and arrest seizures (Stacey and Litt, 2008) or to restore episodic memory (Hampson et al., 2013; Sankar et al., 2014). While there has been tremendous interest in BCIs, including several pilot trials in human patients and an explosion of publications in the past few decades, the clinical benefits have remained quite limited. The purpose of this essay is to address the disconnect between hundreds of laboratories around the world toiling on BCIs and the thousands to millions of patients who could benefit from this technology yet are not.

“Motor” BCIs refer to systems that decode intended movement and use this decoded information to control some object in the world: such as a computer to type out text for communication, turn off and on light switches, navigate with a wheelchair, or control one’s own body with external powered braces or internal neuromuscular stimulators. Given several decades of work on external scalp-EEG based BCIs and nearly a decade since the first human patient was implanted with a multi-electrode array to decode motor intent (Hochberg et al., 2006; Lebedev, 2014), what are the limitations holding back this promising technology from entering the mainstream for clinical care? Sadly, there is no lack of people paralyzed by spinal cord injury, stroke, brain injury, muscular dystrophy and amyotrophic lateral sclerosis. One possible answer would be to counsel patience: while there are over 300,000 people with sensorineural hearing loss who have been implanted with cochlear implants, it has been over seven decades since Djourno and Eyriès showed in 1957, that an inner ear electrode could elicit sound sensations in a deaf listener (Djourno and Eyries, 1957; Macherey and Carlyon, 2014). Is there any way we can learn from prior mistakes and successes so that the translation cycle may be accelerated to bring these therapies to the clinic faster than seven decades?

## Technical limitations

### Inflammation/gliosis

Several groups have demonstrated that motor intent can be decoded from the activity of ensembles of neurons recorded by microelectrode arrays implanted into the neocortex of paralyzed people (Hochberg et al., 2012; Collinger et al., 2013). A problem for chronic recording of single units is that the number and quality of recordings fall off with time (Suner et al., 2005; Barrese et al., 2013; Wang et al., 2014). Several explanations have been posited: the foreign body of the array induces a reactive gliosis with scarring or inflammation, the device experiences micromotion relative to the cell bodies it seeks to record quenching signal to noise, or the device itself fails internally such as through mechanical breakage of electrodes or electrolytic changes in surface chemistry altering impedance (Prasad et al., 2014). Certain groups have attempted to address this device-brain interface problem by altering the microscopic geometry or surface chemistry of the devices (Sanchez et al., 2006; Moxon et al., 2007; Sommakia et al., 2009; Frewin et al., 2011; Ceyssens et al., 2013; Edgington et al., 2013) others have attempted to circumvent the problem entirely by focusing analysis on the envelope of multi-unit activity or analyzing time series in the frequency domain, rather than requiring single units to be discriminated (Dolan et al., 2009; Flint et al., 2013; Lebedev, 2014; Perge et al., 2014).

### Chasing the noise

For motor BCIs there are two learning systems: the mathematical algorithm that decodes neural activity into motor commands, and the patient’s brain itself. Unlike alpha motor neurons in the spinal cord which are “hard-wired” in motor pools to specific sets of skeletal muscles, the relationship of cortical neurons to external muscular and somatosensory features is fluid. If the calibration routines used are too frequent or extreme, then the two learning systems will fail to converge on a decoding-control solution and instead will chase the noise, and the paralyzed patient will not be able make use of the device (Wu et al., 2004). It would be analogous to attempting to learn to ride a bicycle if the laws of physics changed with every attempt to pedal.

### Calibration and technicians

All types of motor BCI, whether non-invasive scalp EEG or direct invasive BCI with implanted electrodes, require considerable set up and calibration with one or more technicians (Sellers et al., 2010; Taherian et al., 2014). Even in the net-connected age with telemedicine, the burden of daily calibration becomes so onerous as to render the motor BCI unfeasible for widespread clinical application (Rupp, 2014).

## Challenging preconceptions

### Preconception: less invasive technology is safer for patients than more invasive technology

Just because a technology does not involve a surgical procedure does not mean it is not risky. The balance of risk and benefit must take into account all aspects of a technology, not simply whether one cuts into the skin. As an example, while the heart can be defibrillated and paced by electrodes worn on the chest, this would be completely impractical for patients to use on a daily basis. The system had to be implanted to make it useful. While motor BCIs may not have proved their utility to an equal degree of implanted cardiac pacemakers, the point is that the focus of device development should be on the overall net utility: the greater the potential benefit, the more a given amount of risk could be taken. In terms of risks, there seems to be significant misunderstanding on the part of non-clinicians about what procedures pose risks to patients and what do not. Relative to other neurosurgical procedures, implantation of tiny microelectrode arrays into the surface cortex is less risky than other common neurosurgical procedure. Likewise, the daily scrubbing of a patient’s scalp and attachment of electrode pads has its own risks of skin breakdown and even fatal cellulitis in patients who have limited mobility and may be in a constant state of relative immunocompromise (Rupp, 2014). Patients with quadriplegia invariably already have undergone surgeries and have percutaneous devices, such as tracheostomy tubes attached to ventilators and feeding tubes. It would be more useful and safer for quadriplegic patients if scalp EEG systems could be rendered as implantable systems: for example, subdermal grids with wireless telemetry. A minimally invasive approach would simultaneously address several bottlenecks in application: it would decrease the risk of skin infection from repeated scalp electrode application, it would decrease impedance variability that affects device performance, and it would take out the reliance on an external technician to physically affix the electrodes every day to afford useful communication. Conclusion: “Degree of invasiveness” is not a helpful metric of neurotechnology safety or utility. Scientists and engineers developing devices should take a holistic view of how the device affects the patient’s overall health.

### Preconception: BCIs will not be clinically useful until they can extract more information

Over the past several decades, several groups working on primate motor neurophysiology have found that the single-unit, ensemble and local field potential activity of motor areas in the brain can be “decoded” to yield information about a wide variety of motor parameters: not only two and three-dimensional end-point (i.e., hand) trajectory, but also muscle contraction states, pattern generator and spinal synergy activation states, joint kinematics, velocity and acceleration, attentional states, sequence and planning features and somatosensory fields (Carpenter et al., 1999; Matsuzaka et al., 2007; Stark et al., 2007; Umilta et al., 2007; Scott, 2008; Zach et al., 2008; Griffin et al., 2009; Vargas-Irwin et al., 2010; Pruszynski et al., 2011; Saleh et al., 2012; Addou et al., 2014; Crowe et al., 2014; Kirsch et al., 2014). Information rate, following the conventions initially developed by Claude Shannon, have been one popular way of quantifying BCI performance (Baranauskas, 2014). While elucidating from a basic neurophysiology perspective, these approaches do not axiomatically translate into device development and clinical utility. Information transfer metrics that elucidate how single neurons transform sensory and motor phenomena (Rieke et al., 1997), tend to devolve into unhelpful distortions of more appropriate performance metrics (such as task completion time or validated daily living functional measures; Peckham et al., 2001). From a practical engineering perspective it would be far better to have one or two degrees of freedom that could be decoded in a fast, reliable, technician-free manner, than seven degrees of freedom that were unreliable and required considerable external supervision to derive (Peckham et al., 2001; Rupp, 2014). While entropy rates can be constructed so as to include reliability as a feature, they are usually not considered in this manner. Another important feature to consider in addition to reliability is subjective effort. In principle, even one’s heart rate could be used as input to a BCI: clearly such a tactic would require considerable mental focus and would be confounded by environmental distractions (e.g., accidentally launching oneself in a heart-rate driven wheelchair upon hearing a horn honk). The farther one moves from the neocortical areas driving voluntary movement, the more challenging it is for a patient to acquire and sustain control. While direct BCIs have been touted for their ability to yield more degrees-of-freedom and signal complexity, their greatest benefit may in fact be the fact that voluntary modulation of signals recorded intracranially from motor areas is most akin to natural movement and hence is subjectively effortless for the human participant, much as it is for healthy humans moving their intact limbs. Conclusion: The quantity and complexity of information are limited metrics for BCI translation to clinical application. BCIs will be clinically useful when they can extract information in a reliable and subjectively effortless manner with minimal calibration or technician supervision.

### Preconception: human pilot trials for direct BCIs must rely on venture capital funding or rare federal opportunities

It usually takes 7 years and costs $40 million to take a final medical device prototype from bench to bedside. It takes two to three years of preparation and nearly $6 million just to launch an initial Investigational Device Exemption (IDE) trial. What exactly does this money pay for? One component is to salary support for consultants with regulatory expertise: this knowledge of the inner workings of the FDA, CE and other foreign equivalents, does not typically “live” inside academia. To have any hope of translational success, investigators must recruit regulatory colleagues who have successful track records of shepherding novel devices through the IDE process. The upfront $6 million also pays for “freezing the design” of the device or fabrication process, establishing clean room Good Manufacturing Practices, and sending off device prototypes to existing, commercial “testing houses” that can systematically test the toxicity and biocompatibility of the device and its electrical safety in the hospital environment. While purposefully incorporating already-approved well-tested materials and fabrication techniques can reassure regulators, these agencies ultimately require these additional tests.

The organizational complexity and financial cost of this process does not fit into the typical R01 or non-NIH equivalents that sustain academic neuroscience laboratories. These leave translational investigators with few options. One is to appeal to special multi-center U01, Veteran’s Affairs or other military-based (e.g., DARPA) multi-million dollar requests for applications. The FES Center in Cleveland, has been successful in following this non-commercial multi-center approach for their neuromuscular stimulator system to restore independent voluntary movement in veterans with spinal cord injury (Peckham et al., 1988). The Department of Defense may withdraw funds if performance metrics aren’t met rendering it difficult to plan appropriately for multi-year trials. While the National Institutes of Health sponsors intramural clinical trials, there is not as extensive a track record for extramural ones devoted to novel devices. In terms of funding academic-industry partnerships, SBIRs and STTRs are simply not at the scale of $6 million needed.

The other option is to create a startup neurotechnology company and apply for funding from angel investors and venture capitalists. Except for very simple mechanical based neurotechnologies (such as a new kind of shunt), few BCI technologies are at a stage of commercialization potential that render them appropriate for risk-averse investors. A pilot trial for a BCI may simply not make any investment sense for the typical venture capital funding model (Ford and Nelsen, 2014). If anything, a promising BCI would be more ripe for VC funding after a pilot trial demonstrated safety and efficacy.

This investigator therefore proposes that federal agencies create new funding mechanisms that fill this gap. These funds would help investigators set up clean room fabrication facilities and cover the cost of the numerous regulatory-required tests for the device. Ideally, all members on the study sections for this putative new mechanism would have some kind of clinical trial expertise, including physicians, FDA regulators, and scientists or engineers who have already successfully run human trials on their own. Given the scale and duration of the funding, and the fact that sudden withdrawal of funding in the middle of a trial could potentially risk patient health, thought should be given to render this new mechanism “sequester-proof” should political forces slash funding. Since industry investors would be the financial beneficiaries of these trials, one approach would be to set aside transparent pools donated by industry explicitly allocated for this novel translational funding mechanism. Agencies could make this financially worthwhile if they could eliminate waste and streamline the process, thus increasing the amount of return for each dollar invested on pilot trial development. By pulling actual FDA regulatory officials into these novel study sections, investigators and future investors would also reap enormous benefit in the regulatory process with this “insider” knowledge. Rather than have each BCI team muddle through the prototype, clean room, standard biosafety/bioelectrical testing, by itself, this mechanism would have its own streamlined process. Conclusion: Leaders in government, industry and academia should forge new funding mechanisms that can help investigators shepherd promising BCI technologies into pilot clinical trials to a stage where traditional existing VC and industry funding make sense and the chances of commercialization were greater.

### Preconception: if a BCI were safe and effective, market forces will automatically propel it towards widespread clinical use

Many good ideas may never end up helping patients due to a variety of reasons as they may not be marketable or may be badly marketed (Vecht et al., 2010). Despite clear demonstration of the safety and efficacy of the FreeHand functional electrical stimulation system to help patients with spinal cord injury (Peckham et al., 1988; Taylor et al., 2002), the small company commercializing it (NeuroControl) went out of business before meeting clinical demand. Scientists seeking to bring a promising neurotechnology from bench to bedside would do well to understand why certain efforts flounder and why others succeed (de Ana et al., 2013; Pisano, 2006; Galloway, 2007; Fletcher and Bourne, 2012). Neurotechnology ventures need to involve business experts early (Leuthardt, 2013) to ensure they can navigate issues of patents, pricing, reimbursement, and multi-year alliances (Pangarkar and Hutmacher, 2003; Bergsland et al., 2014). Inventors must recognize the importance of skilled management (Burns et al., 2009), and have realistic expectations of how commercialization unfolds (Galloway, 2007; Fletcher and Bourne, 2012). Translational scientists must learn that: what drives science does not drive business, there is no single path to commercialization, “research” and “development” are very different phases, the market may not exist at the outset, and that customers are the “ultimate peer review” (Fletcher and Bourne, 2012). Conclusion: While having a strong safety and efficacy profile is necessary for a medical device, it is not sufficient to reach patients in need. To reach patients, devices must be supported by skilled management in both startup and established biotechnology companies.

### Preconception: the market for neuroscience medical device applications is too heterogeneous and small to be worth the investment

Any business from a Fortune 500 company to a corner bodega can be run poorly or well: the fact that there is more than one company manufacturing cochlear implants, for a market of less than a million patients, is testament to the fact that a device with demonstrable safety and efficacy can be financially sustainable. Furthermore, both DBS and cochlear implants are designed to improve quality of life: patients are not expected to die directly from deafness or tremor. Conclusion: To reach and benefit patients, safe and effective technologies must be brought to market by visionary entrepreneurs who have excellent management skills and a deep understanding of the clinical neuroscience landscape.

### Preconception: the brain is too complex: any medical device cannot succeed until the brain is better understood

While cochlear and auditory-brainstem implants leverage neuroanatomical tonotopy to “play” neural structures, the fundamental mechanism of deep brain stimulators for movement disorders remains a source of controversy. Medicine is replete with countless treatments that are used daily to successfully improve human health despite the mechanism of these treatments not being understood. The efficacy of medical interventions is established empirically rather than mechanistically. Hundreds of medications are used to treat brain-based conditions (such as schizophrenia and epilepsy) despite our limited knowledge about the pathophysiology of these conditions or how particular medications exercise their effects. Conclusion: Neurotechnoloogy that can concretely help people can be financially remunerative even despite our incomplete knowledge of the human brain. Companies can “do well by doing good” by focusing on concrete quality of life outcome measures rather than relying on a mechanistic understanding of neurobiology.

### Preconception: the time to approach clinicians and patients when considering human applications of direct BCI or other neurotechnologies is only after the technology itself is finalized and animal studies are completed

Development of the Utah array from a research tool restricted to animal investigation to a clinical intervention in a pilot trial for human patients, benefited from close, friendly collaboration between engineers designing the device within industry and academic neurosurgeons. By literally handing prototypes to experienced surgeons to test in animal models and human cadavers, engineers could gain immediate feedback about helpful or limiting design features that no amount of bench work could reveal (Suner et al., 2005; Som et al., 2014). Conclusion: Scientists should engage physicians, surgeons and patients much earlier in the design cycle.

## Recommendations for improving neurotechnology development

BCI translation can be accelerated by tightening the design cycle with close collaboration between engineers, scientists, surgeons, regulatory experts, and clinicians.

Basic scientists and engineers are urged to never assume what risk-safety profiles are needed for a device to help a person: they should query physicians, surgeons and even potential beneficiaries and their families, sooner rather than later to ascertain the medical context, and should focus their energies on making the device truly useful.

Engineers and scientists are encouraged to visit the patients whose their technology is intended to help and understand what solutions they have deployed now to inform what the new technology must outperform.

Considerations of safety must be holistic and take into account the overall clinical context: non-invasive devices are not necessarily safer or more practical than invasive ones.

While metrics such as “degrees of freedom” and “entropy bit rate” have their utility, to facilitate clinical translation, device developers should focus on increasing device reliability, decreasing subjective effort, reducing calibration and minimizing technician supervision.

Leadership in government and industry are encouraged to consider alternate funding mechanisms that can shepherd technologies farther towards commercialization.

Scientists, surgeons, engineers and physicians seeking to commercialize promising neurotechnologies should recruit entrepreneurs with considerable management skill and a track record of shepherding devices into profitable commercialization.

## Conflict of interest statement

The author declares that the research was conducted in the absence of any commercial or financial relationships that could be construed as a potential conflict of interest.
